# Posttranslational modifications of heterologous proteins expressed in *Nicotiana benthamiana*

**DOI:** 10.1111/pbi.70176

**Published:** 2025-06-08

**Authors:** Kathrin Göritzer, Somanath Kallolimath, Richard Strasser

**Affiliations:** ^1^ Department of Biotechnology and Food Science, Institute of Plant Biotechnology and Cell Biology BOKU University Vienna Austria

**Keywords:** glycosylation, glycoengineering, molecular farming, protease, recombinant protein, tyrosine sulfation

## Abstract

The success of *Nicotiana benthamiana* as a workhorse for heterologous protein production is closely linked to its accessibility and tolerance to genetic manipulation, allowing efficient engineering of posttranslational protein modifications (PTMs) that are critical for the function and stability of heterologous proteins. Therefore, control over PTMs has a significant impact on the quality of a product. Most recombinant protein therapeutics are glycosylated, and glycosylation is the most common and complex PTM. The machinery for initiating N‐glycosylation is largely conserved in *N. benthamiana*, and there are generally fewer glycosyltransferases involved in modifying N‐glycans compared to human cells. This results in less processed and more homogeneous complex N‐glycans, which serve as acceptors for various extensions and the generation of tailored N‐glycans. O‐glycosylation is different and quite diverse in plants. Recent advances in genome editing have resulted in *N. benthamiana* with greatly reduced plant‐specific modifications, making it a valuable tool for studying O‐glycosylation and the production of heterologous proteins with human‐type O‐glycans. In contrast to glycosylation, there are far fewer studies focusing on other PTMs, and the engineering of these modifications in plants is still in its infancy. Noteworthy exceptions include the successful tyrosine sulfation of antibodies and the use of the human protease furin for the activation of recombinant proteins, achieved through a controlled proteolytic processing approach. In summary, recent advances in genome editing and pathway engineering by transient or stable co‐expression of multiple foreign genes in *N. benthamiana* lay the foundation for novel protein‐based products with optimized functions.

## Introduction

Posttranslational modifications (PTM) are crucial for the efficient heterologous production of high‐quality proteins, especially when used as therapeutic proteins or vaccines. Many recombinant proteins, including monoclonal antibodies (mAb), enzymes, hormones, cytokines, coagulation factors, extracellular domains of receptors, and many viral envelope proteins used in VLPs or as soluble vaccine candidates, are typically glycosylated (Eidenberger *et al*., 2023; Göritzer and Strasser, 2021; Margolin *et al*., 2020b; Watanabe *et al*., 2019). As one of the most important PTMs of protein therapeutics, glycosylation affects the folding and therefore has a huge impact on heterologous protein production in any expression system (Helenius and Aebi, 2004). Glycosylation influences protein stability (e.g. degradation and aggregation) and affects the pharmacokinetics of the protein in the human body, the intracellular uptake and their immunogenicity (He *et al*., 2024; Walsh and Jefferis, 2006). More than 200 antibodies have been approved for therapy (Crescioli *et al*., 2025), and most of them are of the IgG1 subclass, which has an N‐glycan at the conserved Asn297 site in the CH2 domain of the heavy chain. This N‐glycan is important for effector functions by modulating the interaction with Fcγ‐receptors (Shields *et al*., 2002; Shinkawa *et al*., 2003). Due to its functional importance in modulating the immune response, therapeutic antibody glycosylation is considered a critical quality attribute that should be within a certain range for optimized efficacy (Reusch and Tejada, 2015; Wang and Ravetch, 2019). Protein glycosylation is a non‐template driven process that is controlled by the amino acid sequence of the protein, the resulting protein conformation and the presence of the glycosylation machinery in the cells.

This review especially focuses on glycoengineering approaches carried out in *N. benthamiana* in recent years. Additional studies are summarized that have been undertaken to modify other PTMs of *N. benthamiana*‐produced proteins, including tyrosine O‐sulfation and controlled proteolytic processing.

## N‐glycosylation

N‐glycosylation is initiated in the lumen of the ER by the transfer of a pre‐assembled oligosaccharide to an asparagine in a specific sequence motif (Asn‐X‐Ser/Thr, X any amino acid except proline) present on a nascent polypeptide chain. In most eukaryotes, including plants, this reaction is catalysed by the multi‐subunit oligosaccharyltransferase (OST) complex (Jeong *et al*., 2018; Strasser, 2016) and the oligosaccharide transfer takes place already while the polypeptide is still translated, which has a direct impact on folding due to the hydrophilic nature of the oligosaccharide (Aebi *et al*., 2010; Hanson *et al*., 2009). In addition to cotranslational modification of sites, a different OST complex mediates posttranslational glycosylation at specific sites in mammals (Ruiz‐Canada *et al*., 2009). In general, not all Asn‐X‐Ser/Thr sites on proteins are glycosylated, and recombinant proteins can display some variation in site occupancy caused by incomplete glycosylation. The attached N‐glycans are subsequently processed by ER‐resident glycosidases, which generate a glycan signal that promotes folding by interacting with the lectins calnexin/calreticulin and associated proteins (Strasser, 2016). Properly folded glycoproteins are released from ER quality control and exit to the Golgi, where the N‐glycans are further processed by α‐mannosidases and glycosyltransferases. This results in the formation of complex‐type N‐glycans that are predominately found on secreted proteins.

## N‐glycosylation site occupancy

**Figure 1:**
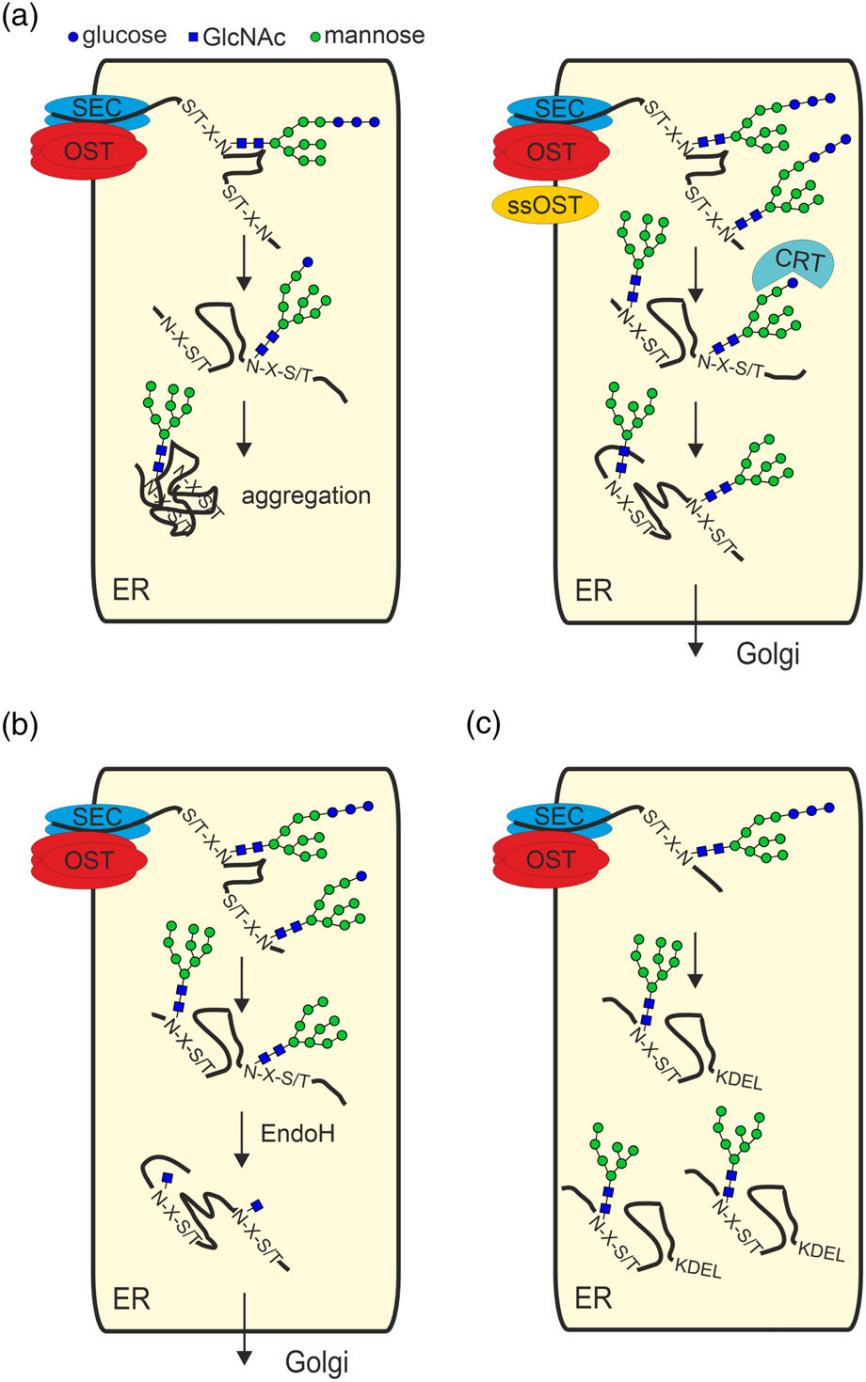
PTM engineering strategies in the endoplasmic reticulum. (a) Protein underglycosylation, caused by incomplete transfer of the pre‐assembled oligosaccharide to N‐X‐S/T sites by the plant oligosaccharyltransferase (OST) complex, can affect folding and increase the propensity of heterologous proteins to aggregate. This can be overcome by co‐expression of a single subunit oligosaccharyltransferase (ssOST) from *Leishmania*, that likely functions independent of the native OST complex. Co‐expression of the ER‐resident lectin chaperone calreticulin (CRT) can further enhance folding efficiency by preventing aggregation of folding intermediates. (b) Co‐expression of endoglycosidase H (EndoH) in the ER results in the formation of glycoproteins carrying a single GlcNAc residue. (c) The presence of a C‐terminal KDEL tetrapeptide, a known Golgi‐to‐ER retrieval signal, results in the accumulation of heterologous glycoproteins with mannosidic N‐glycans (mainly Man_9_GlcNAc_2_ to Man_7_GlcNAc_2_) in the ER. Symbols for monosaccharides are drawn according to the guidelines of the Consortium for Functional Glycomics.

The N‐glycosylation site occupancy of *N. benthamiana*‐expressed heterologous proteins differs, with some sites being unaffected and almost fully glycosylated and other sites from the same protein being partially glycosylated (Castilho *et al*., 2018; Eidenberger *et al*., 2022; Keshvari *et al*., 2024; Montero‐Morales *et al*., 2017; Pisuttinusart *et al*., 2024). Reduced N‐glycosylation site occupancy leads to heterogeneity (macroheterogeneity), which can have various consequences for proteins (Margolin *et al*., 2020b). For example, the removal of one of the two N‐glycans from recombinant SARS‐CoV‐2 RBD negatively influenced the protein production in *N. benthamiana*. However, the expression of single glycosylated RBD could be rescued by co‐expression of human calreticulin, which promotes folding (Shin *et al*., 2021). This is consistent with the known impact of N‐glycosylation on glycoprotein folding in the ER (Aebi *et al*., 2010). Underglycosylation of proteins like IgG1 or IgA2 affects biophysical properties like thermal stability and leads to an increased propensity for aggregation, which has an impact on downstream processing and the overall yield (Göritzer *et al*., 2024; Ruocco *et al*., 2024). Underglycosylation of the tailpiece N‐glycosylation site of IgA1/IgA2 resulted in reduced formation of dimeric IgA1/IgA2 (Göritzer *et al*., 2020), indicating an important role of the tailpiece N‐glycan for joining chain incorporation. Non‐glycosylated IgG1 is known to have no or altered effector functions (Ju and Jung, 2014), and underglycosylation of the Fc site from different *N. benthamiana*‐produced IgG1 affects the Fcγ‐receptor interaction (Beihammer *et al*., 2023; Göritzer *et al*., 2024). Recombinant human IgG1 is typically fully glycosylated at the Asn297 site when produced in mammalian cells. Notably, in plant‐produced cetuximab IgG1 the N‐glycosylation site in the Fab domain is almost fully glycosylated, while the Fc site is only 50–70% glycosylated (Eidenberger *et al*., 2022). This highlights the differences between the plant and mammalian N‐glycosylation machinery and demonstrates the inherent site‐specificity of the plant OST complex. Interestingly, transient expression of IgG using the magnICON vector, which normally gives very high yields (Giritch *et al*., 2006), led to increased amounts of unglycosylated IgG (Eidenberger *et al*., 2022). Similarly, the codon optimization to improve protein expression also led to underglycosylation for the IgG3 Fc site (Sun *et al*., 2023b). This suggests that the activity of the *N. benthamiana* OST complex cannot fully cope with very high protein loads entering the ER. However, there are several approaches to overcome this limitation of the *N. benthamiana* expression platform. In some protists, OST exists as a single catalytic subunit, and transient co‐expression of a single subunit OST (ssOST) from *Leishmania major* (LmSTT3D) or *Leishmania donovani* (LdOST) in *N. benthamiana* resulted in almost full site occupancy on Asn297 in the IgG1 Fc domain and on other recombinant glycoproteins (Beihammer *et al*., 2023; Castilho *et al*., 2018) (Figure 1a). Human virus‐derived glycoproteins can be underglycosylated when transiently expressed in *N. benthamiana*, and this can be in part overcome by LmSTT3D co‐expression (Margolin *et al*., 2022b, 2023; Pantazica *et al*., 2023).

Alternatively, protein engineering can be used to improve the N‐glycosylation of the Fc site on transiently expressed human IgG1. The local amino acid sequence environment of the Asn297 site has been demonstrated to have an impact on N‐glycosylation efficiency (Göritzer *et al*., 2024). A significant improvement in Asn297 site occupancy of mAbs was achieved following mutation of Tyr300 to Leu300, an amino acid that is found at this position in the mouse IgG2 heavy chain.

As outlined above, for many heterologous proteins, the objective is to enhance N‐glycosylation. For certain proteins, however, glycosylation is required for proper folding but is dispensable or even disadvantageous for other functions. In such cases, the complete or partial removal of N‐glycans, leaving no or a single GlcNAc attached to the N‐glycosylation site, is advantageous. The GlycoDelete approach was initially developed in mammalian cells and has also been found to be applicable to Arabidopsis seeds (Meuris *et al*., 2014; Piron *et al*., 2015). A simplified adaptation of the GlycoDelete approach used for deglycosylation involves transient co‐expressing of a single endoglycosidase with the heterologous protein (Figure 1b). This approach allows for the removal of the glycans in the ER and/or Golgi with no or little impact on protein folding and has been used to produce deglycosylated antigens (Mamedov *et al*., 2019, 2021). The endoglycosidase can be targeted to either the ER or the Golgi apparatus, where it is responsible for the removal of the majority of oligomannosidic N‐glycans from recombinant glycoproteins. It is highly probable that the Golgi‐targeted enzyme is already active in the ER. *In planta* deglycosylation using a Golgi‐targeted endoglycosidase H has been shown to result in the *N. benthamiana*‐based production of an ACE2‐Fc receptor decoy carrying single GlcNAc residues attached to its seven N‐glycosylation sites (Izadi *et al*., 2023a). Notably, deglycosylated ACE2‐Fc has been observed to be more potent in SARS‐CoV‐2 virus neutralization than glycosylated ACE2‐Fc (Izadi *et al*., 2023a), highlighting the potential of *in vivo* deglycosylation of heterologous proteins.

## N‐glycan processing and the elimination of unwanted plant‐specific N‐glycan modifications

Upon transfer, the N‐glycans on heterologous proteins are rapidly processed in the ER by α‐glucosidases and α‐mannosidases. Heterologous proteins that are retained or retrieved to the ER, for example by the attachment of a C‐terminal KDEL tetrapeptide, will carry mannosidic N‐glycans (Figure 1c). Many different mAbs with a KDEL fusion at the C‐terminal end of the heavy chain have been produced in *N. benthamiana* (for a recent study see Pisuttinusart *et al*., 2024). While retention in the ER can prevent unwanted proteolytic degradation in the plant secretory pathway and thus increase the overall yield of intact antibodies, the N‐glycans are not processed. The resulting mannosidic N‐glycans are a dead end in terms of glycoengineering, which affects functional properties like effector functions. Antibodies with mannosidic N‐glycans are rapidly cleared from human serum (Goetze *et al*., 2011; Wada *et al*., 2019).

**Figure 2:**
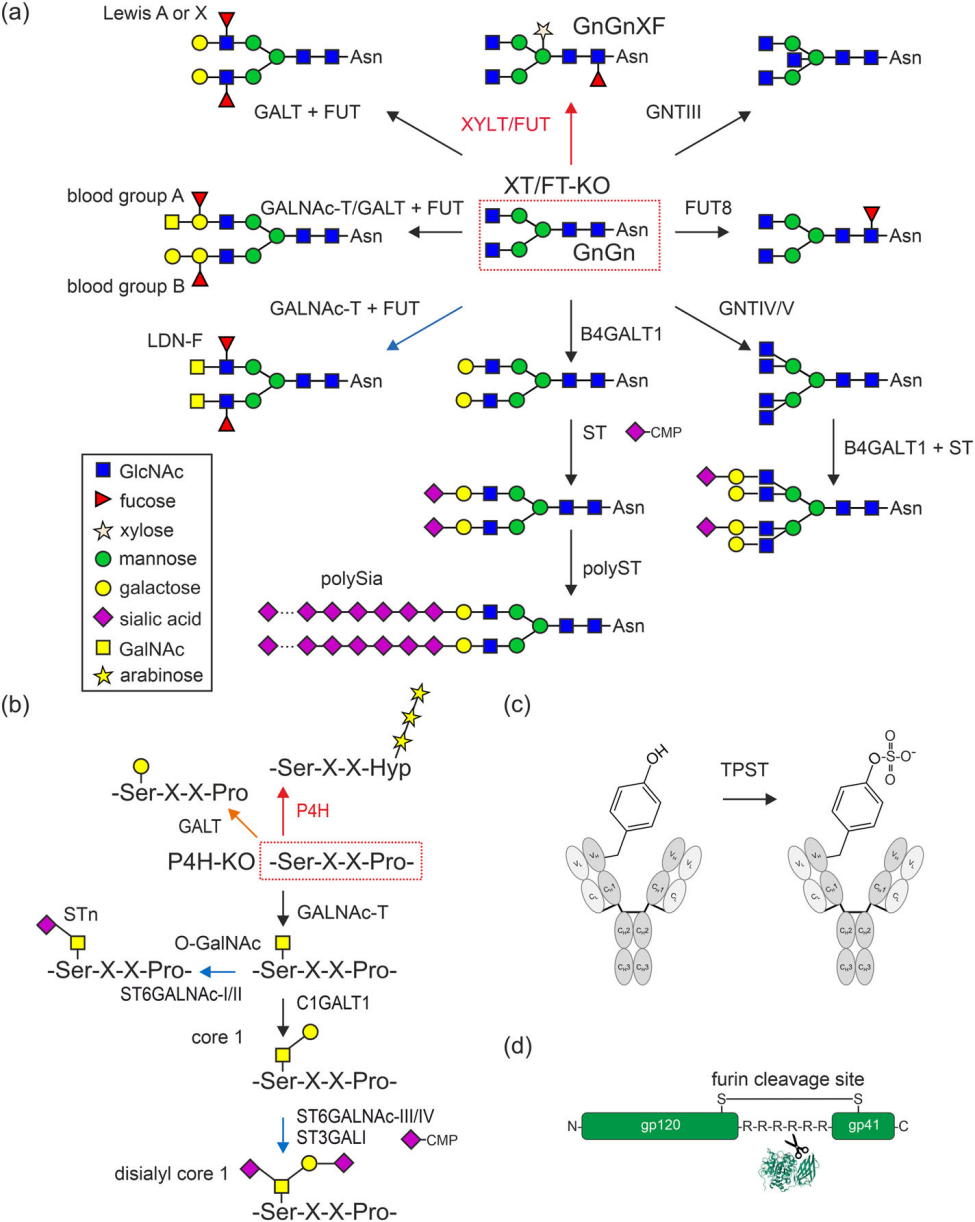
PTM engineering strategies in the Golgi apparatus. (a) Knockout of XYLT/FUT (red arrow) results in the formation of GnGn as the major type of complex N‐glycan (instead of the GnGnXF found in wild‐type plants), which serves as an acceptor substrate for various elongation reactions. FUT, fucosyltransferase; GALT, galactosyltransferase; GALNAc‐T, N‐acetylgalactosaminyltransferase; GNT, N‐acetylglucosaminyltransferase; ST, sialyltransferase; polyST, polysialyltransferase; XYLT, xylosyltransferase. All glycan‐modifying steps indicated by black arrows have been engineered in *N. benthamiana*. The blue arrow indicates a step that has been carried out with wild‐type plants still carrying β1,2‐xylose and core α1,3‐fucose. (b) Knockout of prolyl 4‐hydroxylase (P4H) (red arrow) prevents hydroxyproline (Hyp) formation and modification with arabinose chains. Introduction of the mucin‐type O‐glycosylation pathway results in different mammalian‐type O‐glycans. Glycan‐modifying steps indicated by black arrows have been engineered in *N. benthamiana*. The orange arrow indicates a knockout step that has not been described yet. Blue arrows indicate a step that has been carried out in plants that still produce Hyp. (c) Human tyrosyl protein sulfotransferase (TPST) catalyses the sulfation of specific tyrosine residues in heterologous proteins. (d) Human furin cleaves heterologous proteins at specific sites.

Proteins trafficking through the Golgi are subjected to further processing by α‐mannosidases and several glycosyltransferases. These proteins carry complex‐type N‐glycans (Figure 2a) with GlcNAc_2_Man_3_XylFucGlcNAc_2_ (GnGnXF) as the most prominent form. Incomplete processing or cleavage of terminal GlcNAc residues by β‐hexosaminidases results in the formation of truncated complex N‐glycans (GnMXF, MMXF). The presence of complex and truncated N‐glycans carrying β1,2‐xylose and core α1,3‐fucose has raised concerns because they have been shown to be immunogenic in animal models (Bardor *et al*., 2003; Jin *et al*., 2006) and both residues are part of epitopes that are recognized by anti‐glycan IgE antibodies (Tretter *et al*., 1993; van Ree *et al*., 2000). These concerns prompted the first glycoengineering approach in *N. benthamiana* using gene silencing to get rid of the responsible β1,2‐xylosyl‐ (XYLT) and core α1,3‐fucosyltransferases (FUT) (Strasser *et al*., 2008). The resulting ΔXT/FT line (sometimes called ΔXF) leads to predominantly GnGn N‐glycan structures on secreted glycoproteins and has been used worldwide by academic groups and industry to produce a great panel of different recombinant glycoproteins. Human IgG antibodies produced in ΔXT/FT are virtually devoid of the plant‐specific complex N‐glycan modifications, have been repeatedly used for GMP production for clinical studies, and were employed, for example to produce a mAb cocktail to treat patients infected with Ebola during the 2014 pandemic in West Africa and against different other viruses (Castilho *et al*., 2011a; Föderl‐Höbenreich *et al*., 2025; Hiatt *et al*., 2015; Kallolimath *et al*., 2021; Moore *et al*., 2021; Qiu *et al*., 2014; Swope *et al*., 2022; Yang *et al*., 2023; Zeitlin *et al*., 2011).

Of note, soon after the first IgG mAbs had been produced in ΔXT/FT, it became clear that the N‐glycan profile (mainly GnGn structures, Figure 2a) on the Asn297 site is much more homogenous compared to CHO‐produced antibodies (Moore *et al*., 2021; Nguyen *et al*., 2023; Stadlmann *et al*., 2008; Strasser *et al*., 2008, 2009; Sun *et al*., 2023a). Due to the lack of core fucose, the antibodies were superior in virus neutralization and displayed enhanced effector functions (Castilho *et al*., 2011a; Forthal *et al*., 2010; Grandits *et al*., 2023; Izadi *et al*., 2024; Stelter *et al*., 2020; Yang *et al*., 2023). However, wild‐type *N. benthamiana*‐produced mAbs carrying GnGnXF display abrogated antibody‐dependent enhancement (ADE) and are therefore safer for the development of antibody therapies against flaviviruses than mammalian cell or ΔXT/FT‐produced counterparts (Sun *et al*., 2023c; Yang *et al*., 2023).

More recent genome editing resulted in the generation of full knockout lines of XYLT and FUT (XT/FT‐KO lines) (Jansing *et al*., 2019; Kogelmann *et al*., 2024a). Heterologous proteins produced in these *N. benthamiana* KO lines completely lack β1,2‐xylose and core α1,3‐fucose residues and have been used for the expression of different antibodies (de Taeye *et al*., 2025; Frigerio *et al*., 2022; Jansing *et al*., 2019; Kogelmann *et al*., 2024a) with similar favourable properties as previously described for ΔXT/FT‐derived antibodies. Importantly, the XT/FT‐KO plants grow normally, are fertile, and display the same leaf biomass and protein yield as ΔXT/FT and non‐engineered laboratory strains of *N. benthamiana*. The tolerance towards glycoengineering is an important feature of *N. benthamiana*, because knockout of complex N‐glycan processing glycosyltransferases in rice or *Lotus japonicus* caused developmental abnormalities and problems with seed number or germination (Harmoko *et al*., 2016; Pedersen *et al*., 2017).

Golgi‐resident glycosyltransferases use nucleotide sugars as donor substrates for glycosylation. Apart from the knockout of unwanted glycosyltransferases, another approach is the controlled interference with the biosynthesis of certain nucleotide sugars. To prevent fucosylation of *N. benthamiana*‐produced heterologous proteins, a bacterial GDP‐6‐deoxy‐d‐lyxo‐4‐hexulose reductase (RMD) was transiently co‐expressed (Kogelmann *et al*., 2023). Reduced core fucosylation was detected on N‐glycans from several different recombinant antibodies, showing that this is a fast alternative strategy to produce fucose‐deficient heterologous proteins.

The N‐glycan profiles of viral antigens produced by *N. benthamiana* for use as a potential vaccine candidate show considerable variation. On recombinant soluble extracellular spike or envelope proteins from a diverse range of human viruses produced in *N. benthamiana*, often a high number of mannosidic N‐glycans are present (Margolin *et al*., 2020a, 2023; Song *et al*., 2024). By contrast, spike proteins present in VLPs may carry highly processed plant complex N‐glycans, including Lewis A structures (Balieu *et al*., 2022). Other *N. benthamiana*‐produced viral antigens, such as the SARS‐CoV‐2 RBD and a chimeric human Hepatitis B virus (HBV) antigen displayed typical plant‐type complex N‐glycans (Pantazica *et al*., 2023; Shin *et al*., 2021). The HBV antigen was produced in WT and XT/FT‐KO plants and displayed considerable amounts of glycans that correspond to galactosylated complex N‐glycans. These glycans were more prominent in XT/FT‐KO and likely carry terminal β1,3‐linked galactose as found in Lewis A structures (Pantazica *et al*., 2023). The variation in N‐glycan processing known as microheterogeneity depends on the glycosylation machinery of the host cell including the availability of nucleotide sugar donor, as well as intrinsic features of the protein that enhance or block the access of N‐glycans to Golgi‐resident processing enzymes. Additionally, non‐canonical secretion with Golgi bypassing may lead to incompletely processed N‐glycans, and the presence of glycosyl hydrolases such as β‐hexosaminidases and β‐galactosidases in the secretory pathway or the apoplast affects the homogeneity of the produced glycans (Kriechbaum *et al*., 2020; Shin *et al*., 2017).

In the context of glycosylated viral antigens, it is virtually impossible to predict the most beneficial glycan structure for the development of vaccine candidates. For instance, the presence of immunogenic sugar residues could function as an adjuvant, thereby amplifying the immune response. Conversely, they may also exert an adverse effect by eliciting an antibody response directed predominantly against the glycans rather than the virus protein itself. The presence of extensive glycan modifications has the potential to mask certain polypeptide epitopes, thereby diminishing a specific immune response. Furthermore, many immunization studies are conducted in animals that exhibit variation in anti‐glycan immune response towards glycosylated proteins (Bardor *et al*., 2003; Jin *et al*., 2006), and the clinical relevance of these findings remains uncertain. Only a limited number of studies have directly compared the immunogenicity of different plant‐derived glycosylation variants. A recent study found that immunizing mice with three different glycosylation variants (main N‐glycan: mannosidic, GnGnXF, GnGn, respectively) of an RBD‐Fc fusion protein resulted in similar anti‐RBD and SARS‐CoV‐2 neutralizing antibody responses (Srisangsung *et al*., 2024). Another study revealed that the antisera derived from mice immunized with an HBV antigen produced in XT/FT‐KO exhibited significantly more potent virus‐neutralizing antibodies compared to the antigen derived from WT (Pantazica *et al*., 2023).

## Engineering of animal and human‐type N‐glycan modifications

In mammalian cells, the GnGn core structure of complex N‐glycans is further elongated by the incorporation of β1,4‐linked galactose and sialic acid (α2,6‐ or α2,3‐linked Neu5Ac). The core GlcNAc is often modified with a α1,6‐fucose, and different N‐acetylglucosaminyltransferases (e.g. GnTIV, GnTV) initiate the formation of branching (Figure 2a). All these complex N‐glycan modifications are absent in plants (Wilson *et al*., 2001), but have been engineered in *N. benthamiana*, and the glycan structures found on recombinant proteins displayed N‐glycans that resembled their mammalian counterparts (Castilho *et al*., 2010, 2011a,b; Kallolimath *et al*., 2016; Strasser *et al*., 2009). While engineering of modifications like core α1,6‐fucosylation is straightforward, for other glycosyltransferases like β1,4‐galactosyltransferase, targeting to a late Golgi and optimal expression level is crucial to prevent the premature attachment of a galactose and generation of hybrid‐type N‐glycans caused by a block of the endogenous processing enzymes (Kallolimath *et al*., 2018; Nguyen *et al*., 2023; Strasser *et al*., 2009; Vézina *et al*., 2009). For stable engineering of β1,4‐galactosylation, the expression level is critical, as too high expression is associated with adverse growth phenotypes (Schneider *et al*., 2015). Recently, an industrial‐grade plant line for the expression of therapeutically interesting proteins that carry β1,4‐galactosylated N‐glycans and lack core fucose and xylose was generated. This line robustly produced mAbs and did not display any severe growth or development phenotype apart from a reduced seed production (Kogelmann *et al*., 2024b). In light of recent findings that galactosylated/non‐fucosylated IgG‐Fcs exhibit enhanced immunomodulatory effects (Mimura *et al*., 2022), this *N. benthamiana* expression platform is particularly interesting. In other studies, β1,3‐galactosylated complex N‐glycans were generated, and fucose was attached in different linkages to produce Lewis‐type glycan modifications, blood group antigens, or fucosylated LacDiNAc extensions (König‐Beihammer *et al*., 2022; Nguyen *et al*., 2023; Schwestka *et al*., 2021; Wilbers *et al*., 2017; Zwanenburg *et al*., 2023) (Figure 2a).

Sialylation is important for capping of terminal galactose residues, which can prevent the efficient interaction with the asialoglycoprotein receptor and thus contribute to an increased half‐life of therapeutic proteins in human serum (Egrie *et al*., 2003). In addition, these negatively charged sugar residues (i.e. N‐acetylneuraminic acid) play essential roles in many aspects of life including cell–cell interactions or signalling (Schnaar *et al*., 2014). Engineering of sialylation in plants is highly demanding because it requires the introduction of the biosynthesis pathway for CMP‐sialic acid, the nucleotide sugar donor, for the sialyltransferases in the Golgi, and a transporter to transport CMP‐sialic acid across the membrane into the Golgi lumen (Castilho *et al*., 2010). Despite these challenges, the coordinated expression of the pathway in *N. benthamiana* resulted in the sialylation of many different recombinant proteins (Castilho *et al*., 2015; Göritzer *et al*., 2019; Izadi *et al*., 2023b; Kallolimath *et al*., 2016, 2020; Montero‐Morales *et al*., 2017).

A unique feature of sialic acid is the formation of linear homo‐polymers in α2,8‐ or α2,9‐linkages. Its most complex form, polysialic acid (polySia), is biocompatible, non‐immunogenic, biodegradable and highly hydrophilic. Hence, its application has become increasingly prominent in drug development and therapeutics. For example, polySia can be used as an alternative to PEGylation to increase the half‐life of drugs (Meng *et al*., 2018), to enhance biodistribution without the risk of toxicity (Zhang *et al*., 2023) or to facilitate the crossing of the blood–brain barrier (Wang *et al*., 2016). Functionally active polysialylated proteins were produced in *N. benthamiana* by co‐expression of the sialic acid pathway with human α2,8‐polysialyltransferases (Kallolimath *et al*., 2016).

## Elimination of plant‐specific hydroxyproline residues

While the N‐glycosylation and N‐glycan processing machinery is partly conserved between all eukaryotes, O‐glycosylation of plants and mammals is fundamentally different (Bennett *et al*., 2012; Petersen *et al*., 2021; Strasser, 2016). In mammals, the most prevalent O‐glycan formation (mucin‐type O‐glycosylation) on secretory proteins involves the incorporation of an N‐acetylgalactosamine (GalNAc) to serine or threonine residues (Bennett *et al*., 2012). The O‐GalNAc is further modified by the stepwise attachment of different monosaccharides, such as galactose, GlcNAc, sialic acid and fucose. This biosynthetic pathway gives rise to a variety of mucin‐type core O‐glycan structures that play crucial roles in numerous biological processes (Thompson and Wakarchuk, 2022; Wandall *et al*., 2021). For many recombinant human proteins, the functional role of O‐glycans is still not very well characterized and glycoengineering will contribute to advance our understanding and develop improved therapies.

In plants, specific serine residues within proteins, such as extensins, undergo a modification involving the addition of a single galactose residue. A more abundant type of PTM is the irreversible conversion of proline to hydroxyproline (Hyp) residues (Petersen *et al*., 2021; Strasser, 2016), which is catalysed by plant prolyl 4‐hydroxylases (P4H) (Figure 2b). On plant‐produced heterologous proteins, these Hyp modifications frequently occur on prolines that appear in small clusters next to mammalian O‐glycosylation sites. The Hyp residues can be further modified by the incorporation of arabinose residues, resulting in the formation of glycoproteins that contain characteristic plant O‐glycans, which are composed of unbranched chains of up to five arabinoses (Karnoup *et al*., 2005). Abundant Hyp modifications and attached pentoses have been detected in the hinge region of recombinant IgA1 (Dicker *et al*., 2016; Uetz *et al*., 2022, 2024) and on mucin‐type peptides (Pinkhasov *et al*., 2011; Yang *et al*., 2012) produced in *N. benthamiana*. The impact of these Hyp residues and attached glycans remains to be elucidated. However, these plant‐specific modifications have been shown to contribute to product heterogeneity, interfere with mucin‐type O‐glycosylation engineering and potentially compromise the safety of heterologous proteins by contributing to immunogenicity. Consequently, multiplex genome editing was used to knockout members of two *P4H* gene families from *N. benthamiana*. While the knockout of all four *P4H4* genes did not markedly reduce the amount of Hyp residues in the hinge region of an expressed IgA1 (Uetz *et al*., 2022), the knockout of two *P4H10* genes drastically reduced Hyp modifications and only small amounts of attached pentoses were detected (Uetz *et al*., 2024). This suggests that P4H10 significantly contributes to Hyp formation on recombinant proteins produced in *N. benthamiana*. Since P4H enzymes display some overlapping substrate specificities (Mócsai *et al*., 2021), the knockout of other members of the P4H family is required to completely eliminate this PTM from recombinant proteins produced in plants. The P4H targets must be carefully selected, as it is known that the knockout of different family members results in plant developmental phenotypes (Velasquez *et al*., 2011).

## Engineering of mammalian‐type O‐glycan modifications

To initiate the generation of human mucin‐type O‐glycans, it is necessary to express a polypeptide GalNAc‐transferase in plants that catalyses the transfer of a GalNAc residue to serine/threonine. In humans, there is a family of up to 20 polypeptide GalNAc‐transferases that control the initiation of mucin‐type O‐glycosylation on proteins (Bennett *et al*., 2012). In addition to the polypeptide GalNAc‐transferases, the expression of a heterologous GlcNAc‐epimerase and GlcNAc/GalNAc‐Golgi transporter has been shown to increase the efficiency of GalNAc transfer (Castilho *et al*., 2012). Transient expression of GalNAc‐transferase 2 and 4 in *N. benthamiana* has been shown to result in the production of GalNAc‐containing recombinant glycoproteins, including erythropoietin (EPO), granulocyte colony stimulating factor (GCSF), mucin domains and IgA1 (Castilho *et al*., 2012; Daskalova *et al*., 2010; Dicker *et al*., 2016; Ramirez‐Alanis *et al*., 2018; Uetz *et al*., 2024; Yang *et al*., 2012). The co‐expression of a β1,3‐galactosyltransferase from *Drosophila melanogaster* resulted in the formation of core 1, and the expression of different sialyltransferases in sialyl core 1, disialyl core 1 and STn antigen formation (Figure 2b) (Castilho *et al*., 2012; Dicker *et al*., 2016; Uetz *et al*., 2024). To date, the engineering of other mucin‐type O‐glycan extensions and other types of O‐glycosylation (e.g. O‐fucose, O‐glucose, O‐mannose, O‐GlcNAc), which are rare on human proteins, has not yet been achieved in plants.

## Other posttranslational modifications of heterologous proteins

In comparison to glycosylation and its various effects on proteins, there is a paucity of knowledge regarding the impact of other plant‐made PTMs on the product's quality. For instance, multi‐component proteins, including mAbs, require the correct formation of intrachain and interchain disulphide bonds for their correct conformation and assembly. Aberrant disulphide bonds may increase the tendency for aggregation of glycoproteins (Hebert *et al*., 1996). Despite the availability of robust MS‐based analytical methods to elucidate the formation of disulphide bonds (Gupta *et al*., 2023), this PTM is often neglected during product characterization. For *N. benthamiana*‐produced human IgA2, it has been suggested that the absence of an additional disulphide bond in recombinant IgA2(m1) has an impact on the thermal stability of the antibody (Göritzer *et al*., 2017). Human protein disulphide isomerase (ERp44) expression did not alter the dimeric IgA1/2 production (Göritzer *et al*., 2020), and *N. benthamiana* ERp57 expression did not improve the production of HIV SOSIP envelope trimers (Rosenberg *et al*., 2022).

ER‐resident chaperons, like calnexin and calreticulin, are known to promote the folding of glycoproteins and, with their interaction partner ERp57, they contribute to the suppression of non‐native disulphide bonds (Parodi, 2000). Human calreticulin co‐expression has been used in *N. benthamiana* to improve the expression of viral glycoproteins (Margolin *et al*., 2020a, 2022a,b, 2023; Rosenberg *et al*., 2022; Shin *et al*., 2021) (Figure 1a) and heterologous expression of Arabidopsis calnexin boosted the expression of fully assembled SIgA (Göritzer *et al*., 2025). Whether the beneficial impact of calreticulin/calnexin was mainly due to the lectin binding function or also mediated by an interacting plant ERp57 orthologue remains to be shown.

## Tyrosine O‐sulfation

Tyrosine O‐sulfation is a PTM that is characteristic of a limited number of human proteins and is found, for example in the complementarity determining region (CDR) of mAbs directed against viruses like HIV (Huang *et al*., 2004). Tyrosine sulfation, the transfer of a sulfuryl group to certain tyrosine residues, is carried out by tyrosyl protein sulfotransferases (TPST), which are Golgi‐resident type II transmembrane proteins (Figure 2c). *N. benthamiana* is not capable of tyrosine O‐sulfation on antibodies. However, this PTM can be introduced by co‐expression of human TPST1 targeted to the late Golgi (Loos *et al*., 2015). Tyrosine O‐sulfation has been found to enhance protein–protein interactions and increase the potency and breadth of neutralizing anti‐HIV antibodies produced in *N. benthamiana* (Loos *et al*., 2015; Singh *et al*., 2020).

## Controlled proteolytic processing – furin‐mediated cleavage

Unintended proteolytic processing of plant‐produced recombinant proteins is a severe issue that affects the yield and quality of recombinant proteins (Beritza *et al*., 2024; Grosse‐Holz *et al*., 2018; Puchol Tarazona *et al*., 2021; Singh *et al*., 2022). It also contributes to protein heterogeneity, for example by clipping the terminal lysine residue from the C‐terminus of the IgG1 heavy chain (Castilho *et al*., 2018).

On the other hand, the initiation of controlled proteolytic processing is a useful tool to modify or specifically activate target proteins. This can be done either by incorporating protease cleavage sites for proteases that are absent in plants. These sites on the recombinant proteins can then be used for *in vitro* processing (Jung *et al*., 2022), for example during downstream processing, or to facilitate *in vivo* processing in specific human tissues (Hou *et al*., 2022). Another approach is the *in planta* co‐expression of a non‐plant protease with the heterologous protein (Figure 2d). Several studies have used co‐expression of the Golgi‐targeted human furin protease in *N. benthamiana* to enhance the expression and activate recombinant proteins such as transforming growth factor beta (Wilbers *et al*., 2016), factor IX (Mamedov *et al*., 2019), and soluble viral glycoproteins such as HIV gp140 (Margolin *et al*., 2020a) or the SARS‐CoV‐2 spike protein (Margolin *et al*., 2022a).

## Protein lipidation

Many human proteins are modified by the covalent attachment of various types of lipid moieties (Yuan *et al*., 2024). Common lipid modifications in recombinant proteins include S‐palmitoylation (or S‐acylation), N‐myristoylation, S‐prenylation or the attachment of a glycosylphosphatidylinositol (GPI) anchor. These lipid modifications significantly increase the hydrophobicity of proteins, resulting in conformational changes, prolonged half‐life, for example through association with albumin in the bloodstream, altered membrane affinity or immunogenicity (Menacho‐Melgar *et al*., 2019). While plants support native‐like lipidation such as GPI‐anchor attachment (Beihammer *et al*., 2020), N‐myristoylation and S‐acylation (Boyle *et al*., 2016), little has been reported on strategies to engineer these or non‐native lipidations on recombinant proteins. This is complicated by the fact that the attachment of many fatty acid moieties takes place in the cytosolic environment, whereas many recombinant proteins are secreted proteins that require the endomembrane system for protein folding and maturation. Therefore, lipidated recombinant therapeutic proteins or peptides are usually produced by chemical conjugation of the lipid (Ramírez‐Andersen *et al*., 2018).

## Conclusion and outlook

Advances in stable and transient pathway engineering in *N. benthamiana* resulted in the production of heterologous proteins with tailored PTMs and improved properties. Subsequent endeavours will address the present limitations, including underglycosylation at particular sites. Optimized N‐glycosylation will not only reduce the heterogeneity but also promote folding and reduce the aggregation propensity, which should result in an overall increased productivity. This will contribute to make *N. benthamiana* economically even more interesting for industry (Ridgley *et al*., 2023). Current heterogeneity associated with N‐glycan processing will be addressed by multiplex genome editing targeting modifications such as β1,3‐galactose and α1,4‐fucose, which are rare in plants but present on some endogenous and *N. benthamiana*‐produced recombinant proteins, including EPO and SARS‐CoV‐2 VLPs (Balieu *et al*., 2022; Beihammer *et al*., 2021; Castilho *et al*., 2013). In addition, glycosyl hydrolase activities (β‐hexosaminidases and β‐galactosidases) that interfere with glycoengineering by cleavage of terminal sugar residues and thus contribute to increased N‐ and O‐glycan heterogeneity should be eliminated in these lines (Kriechbaum *et al*., 2020; Shin *et al*., 2017; van der Kaaij *et al*., 2025). Further characterization of the large P4H family in *N. benthamiana* will identify additional targets for the elimination of Hyp modifications and attached sugar residues from recombinant glycoproteins such as human IgA1 or EPO (Mócsai *et al*., 2021). These multiple knockout lines with simultaneously engineered N‐ and O‐glycans will serve as an optimal expression platform for a variety of recombinant mammalian glycoproteins. Further modifications can then be made by transient or stable expression of relevant non‐plant pathways without interference from plant‐specific glycosylation (Dicker *et al*., 2016; Kallolimath *et al*., 2016; Uetz *et al*., 2024). In addition to whole plants, glycoengineered *N. benthamiana* cell‐based platforms can be used for scalable and transient glycoprotein expression (Dianatkhah *et al*., 2025).

Furthermore, it is anticipated that novel engineering strategies are being developed that, for example alter ER morphology, ER quality control or more generally reduce stress‐dependent pathways associated with Agrobacterium‐mediated recombinant protein expression (Göritzer *et al*., 2025; Hamel *et al*., 2024; Wagner *et al*., 2024). These approaches have implications for PTMs and folding or assembly, as shown for IgA when the ER morphology is altered (Göritzer *et al*., 2025). In addition to the ER, similar approaches could also aim to engineer the Golgi milieu to alter complex N‐glycan processing or downstream compartments where the recombinant proteins accumulate.

Finally, advances in analytical methods, such as intact mass spectrometry (Beihammer *et al*., 2023; Castilho *et al*., 2018; Schachner *et al*., 2024) of recombinant proteins produced in plants, will likely reveal more heterogeneity caused by PTMs that require some attention. Unintended PTMs can be the result of side effects of engineering approaches as observed for glycosylation in *N. benthamiana* (Kogelmann *et al*., 2023) and moss (Bohlender *et al*., 2022). It is reasonable to predict that the combination of engineering tools developed over the last decade and the increased use of genome editing will overcome any such limitations. Emerging applications, such as engineering recombinant protein therapeutics for transfer to the brain or oral administration of drugs, will require novel approaches like the engineering of attached glycan polymers (polySia) (Kallolimath *et al*., 2016) for tissue penetration or proteins for encapsulation (Schwestka *et al*., 2023). Together, these advancements will fuel new developments for heterologous protein production in *N. benthamiana*. Given its inherent capacity for PTM engineering, it is anticipated that the relevance of *N. benthamiana* as an expression platform will continue to experience a marked increase.

## Conflict of interest

The authors have not declared a conflict of interest.

## Disclosure

KG, SK and RS are named inventors of patent applications filed by and assigned to BOKU University.

## Author contributions

Writing original draft: RS. Editing and revisions: KG, SK.

## Data Availability

Data sharing is not applicable to this article as no new data were created or analysed in this study.

## References

[pbi70176-bib-0001] Aebi, M. , Bernasconi, R. , Clerc, S. and Molinari, M. (2010) N‐glycan structures: recognition and processing in the ER. Trends Biochem. Sci. 35, 74–82. 19853458 10.1016/j.tibs.2009.10.001

[pbi70176-bib-0002] Balieu, J. , Jung, J.W. , Chan, P. , Lomonossoff, G.P. , Lerouge, P. and Bardor, M. (2022) Investigation of the N‐glycosylation of the SARS‐CoV‐2 S protein contained in VLPs produced in *Nicotiana benthamiana* . Molecules, 27, 5119. 36014368 10.3390/molecules27165119 PMC9412417

[pbi70176-bib-0003] Bardor, M. , Faveeuw, C. , Fitchette, A. , Gilbert, D. , Galas, L. , Trottein, F. , Faye, L. *et al*. (2003) Immunoreactivity in mammals of two typical plant glyco‐epitopes, core alpha(1,3)‐fucose and core xylose. Glycobiology, 13, 427–434. 12626420 10.1093/glycob/cwg024

[pbi70176-bib-0006] Beihammer, G. , König‐Beihammer, J. , Kogelmann, B. , Ruocco, V. , Grünwald‐Gruber, C. , D'Aoust, M.A. , Lavoie, P.O. *et al*. (2023) An oligosaccharyltransferase from *Leishmania donovani* increases the N‐glycan occupancy on plant‐produced IgG1. Front. Plant Sci. 14, 1233666. 37615026 10.3389/fpls.2023.1233666 PMC10442823

[pbi70176-bib-0004] Beihammer, G. , Maresch, D. , Altmann, F. and Strasser, R. (2020) Glycosylphosphatidylinositol‐anchor synthesis in plants: a glycobiology perspective. Front. Plant Sci. 11, 611188. 33312189 10.3389/fpls.2020.611188 PMC7704450

[pbi70176-bib-0005] Beihammer, G. , Maresch, D. , Altmann, F. , Van Damme, E.J.M. and Strasser, R. (2021) Lewis A glycans are present on proteins involved in cell wall biosynthesis and appear evolutionarily conserved among natural. Front. Plant Sci. 12, 630891. 33777069 10.3389/fpls.2021.630891 PMC7991798

[pbi70176-bib-0007] Bennett, E.P. , Mandel, U. , Clausen, H. , Gerken, T.A. , Fritz, T.A. and Tabak, L.A. (2012) Control of mucin‐type O‐glycosylation: a classification of the polypeptide GalNAc‐transferase gene family. Glycobiology, 22, 736–756. 22183981 10.1093/glycob/cwr182 PMC3409716

[pbi70176-bib-0008] Beritza, K. , Buscaill, P. , Song, S.J. , Jutras, P.V. , Huang, J. , Mach, L. , Dong, S. *et al*. (2024) SBT5.2s are the major active extracellular subtilases processing IgG antibody 2F5 in the *Nicotiana benthamiana* apoplast. Plant Biotechnol. J. 22, 2808–2810. 38852164 10.1111/pbi.14406 PMC11536445

[pbi70176-bib-0009] Bohlender, L.L. , Parsons, J. , Hoernstein, S.N.W. , Bangert, N. , Rodriguez‐Jahnke, F. , Reski, R. and Decker, E.L. (2022) Unexpected arabinosylation after humanization of plant protein N‐glycosylation. Front. Bioeng. Biotechnol. 10, 838365. 35252146 10.3389/fbioe.2022.838365 PMC8894861

[pbi70176-bib-0010] Boyle, P.C. , Schwizer, S. , Hind, S.R. , Kraus, C.M. , De la Torre Diaz, S. , He, B. and Martin, G.B. (2016) Detecting N‐myristoylation and S‐acylation of host and pathogen proteins in plants using click chemistry. Plant Methods, 12, 38. 27493678 10.1186/s13007-016-0138-2 PMC4972946

[pbi70176-bib-0017] Castilho, A. , Beihammer, G. , Pfeiffer, C. , Göritzer, K. , Montero‐Morales, L. , Vavra, U. , Maresch, D. *et al*. (2018) An oligosaccharyltransferase from *Leishmania major* increases the N‐glycan occupancy on recombinant glycoproteins produced in *Nicotiana benthamiana* . Plant Biotechnol. J. 16, 1700–1709. 29479800 10.1111/pbi.12906 PMC6131413

[pbi70176-bib-0012] Castilho, A. , Bohorova, N. , Grass, J. , Bohorov, O. , Zeitlin, L. , Whaley, K. , Altmann, F. *et al*. (2011a) Rapid high yield production of different glycoforms of Ebola virus monoclonal antibody. PLoS One, 6, e26040. 22039433 10.1371/journal.pone.0026040 PMC3200319

[pbi70176-bib-0013] Castilho, A. , Gattinger, P. , Grass, J. , Jez, J. , Pabst, M. , Altmann, F. , Gorfer, M. *et al*. (2011b) N‐glycosylation engineering of plants for the biosynthesis of glycoproteins with bisected and branched complex N‐glycans. Glycobiology, 21, 813–823. 21317243 10.1093/glycob/cwr009 PMC3091529

[pbi70176-bib-0016] Castilho, A. , Gruber, C. , Thader, A. , Oostenbrink, C. , Pechlaner, M. , Steinkellner, H. and Altmann, F. (2015) Processing of complex N‐glycans in IgG Fc‐region is affected by core fucosylation. MAbs, 7, 863–870. 26067753 10.1080/19420862.2015.1053683 PMC4622071

[pbi70176-bib-0014] Castilho, A. , Neumann, L. , Daskalova, S. , Mason, H.S. , Steinkellner, H. , Altmann, F. and Strasser, R. (2012) Engineering of Sialylated Mucin‐type O‐Glycosylation in Plants. J. Biol. Chem. 287, 36518–36526. 22948156 10.1074/jbc.M112.402685 PMC3476317

[pbi70176-bib-0015] Castilho, A. , Neumann, L. , Gattinger, P. , Strasser, R. , Vorauer‐Uhl, K. , Sterovsky, T. , Altmann, F. *et al*. (2013) Generation of biologically active multi‐sialylated recombinant human EPOFc in plants. PLoS One 8, e54836. 23372778 10.1371/journal.pone.0054836 PMC3555983

[pbi70176-bib-0011] Castilho, A. , Strasser, R. , Stadlmann, J. , Grass, J. , Jez, J. , Gattinger, P. , Kunert, R. *et al*. (2010) In planta protein sialylation through overexpression of the respective mammalian pathway. J. Biol. Chem. 285, 15923–15930. 20305285 10.1074/jbc.M109.088401 PMC2871460

[pbi70176-bib-0018] Crescioli, S. , Kaplon, H. , Wang, L. , Visweswaraiah, J. , Kapoor, V. and Reichert, J.M. (2025) Antibodies to watch in 2025. MAbs, 17, 2443538. 39711140 10.1080/19420862.2024.2443538 PMC12952251

[pbi70176-bib-0019] Daskalova, S.M. , Radder, J.E. , Cichacz, Z.A. , Olsen, S.H. , Tsaprailis, G. , Mason, H. and Lopez, L.C. (2010) Engineering of *N. benthamiana* L. plants for production of N‐acetylgalactosamine‐glycosylated proteins‐towards development of a plant‐based platform for production of protein therapeutics with mucin type O‐glycosylation. BMC Biotechnol. 10, 62. 20735851 10.1186/1472-6750-10-62 PMC2936419

[pbi70176-bib-0122] de Taeye, S.W. , Faye, L. , Morel, B. , Schriek, A.I. , Umotoy, J.C. , Yuan, M. , Kuzmina, N.A. *et al*. (2025) Plant‐produced SARS‐CoV‐2 antibody engineered towards enhanced potency and in vivo efficacy. Plant Biotechnol. J. 23, 4–16. 39563066 10.1111/pbi.14458 PMC11672753

[pbi70176-bib-0020] Dianatkhah, S. , Kogelmann, B. , Melnik, S. , Eminger, F. , Kallolimath, S. , Sun, L. , Sumesgutner, D. *et al*. (2025) A plant cell‐based platform for the expression of complex proteins with fucose‐reduced sialylated N‐glycans. Plant Biotechnol. J. 10.1111/pbi.70044 PMC12854884 40207905

[pbi70176-bib-0021] Dicker, M. , Tschofen, M. , Maresch, D. , König, J. , Juarez, P. , Orzaez, D. , Altmann, F. *et al*. (2016) Transient glyco‐engineering to produce recombinant IgA1 with defined N‐ and O‐glycans in plants. Front. Plant Sci. 7, 18. 26858738 10.3389/fpls.2016.00018 PMC4731523

[pbi70176-bib-0022] Egrie, J.C. , Dwyer, E. , Browne, J.K. , Hitz, A. and Lykos, M.A. (2003) Darbepoetin alfa has a longer circulating half‐life and greater in vivo potency than recombinant human erythropoietin. Exp. Hematol. 31, 290–299. 12691916 10.1016/s0301-472x(03)00006-7

[pbi70176-bib-0023] Eidenberger, L. , Eminger, F. , Castilho, A. and Steinkellner, H. (2022) Comparative analysis of plant transient expression vectors for targeted N‐glycosylation. Front. Bioeng. Biotechnol. 10, 1073455. 36619384 10.3389/fbioe.2022.1073455 PMC9812561

[pbi70176-bib-0024] Eidenberger, L. , Kogelmann, B. and Steinkellner, H. (2023) Plant‐based biopharmaceutical engineering. Nat. Rev. Bioeng. 1, 426–439. 37317690 10.1038/s44222-023-00044-6 PMC10030082

[pbi70176-bib-0025] Föderl‐Höbenreich, E. , Izadi, S. , Hofacker, L. , Kienzl, N.F. , Castilho, A. , Strasser, R. , Tarrés‐Freixas, F. *et al*. (2025) An ACE2‐Fc decoy produced in glycoengineered plants neutralizes ancestral and newly emerging SARS‐CoV‐2 variants and demonstrates therapeutic efficacy in hamsters. Sci. Rep. 15, 11307. 40175560 10.1038/s41598-025-95494-w PMC11965572

[pbi70176-bib-0026] Forthal, D.N. , Gach, J.S. , Landucci, G. , Jez, J. , Strasser, R. , Kunert, R. and Steinkellner, H. (2010) Fc‐glycosylation influences Fcγ receptor binding and cell‐mediated anti‐HIV activity of monoclonal antibody 2G12. J. Immunol. 185, 6876–6882. 21041724 10.4049/jimmunol.1002600

[pbi70176-bib-0027] Frigerio, R. , Marusic, C. , Villani, M.E. , Lico, C. , Capodicasa, C. , Andreano, E. , Paciello, I. *et al*. (2022) Production of two SARS‐CoV‐2 neutralizing antibodies with different potencies in *Nicotiana benthamiana* . Front. Plant Sci. 13, 956741. 36131799 10.3389/fpls.2022.956741 PMC9484322

[pbi70176-bib-0028] Giritch, A. , Marillonnet, S. , Engler, C. , van Eldik, G. , Botterman, J. , Klimyuk, V. and Gleba, Y. (2006) Rapid high‐yield expression of full‐size IgG antibodies in plants coinfected with noncompeting viral vectors. Proc. Natl. Acad. Sci. U. S. A. 103, 14701–14706. 16973752 10.1073/pnas.0606631103 PMC1566189

[pbi70176-bib-0029] Goetze, A.M. , Liu, Y.D. , Zhang, Z. , Shah, B. , Lee, E. , Bondarenko, P.V. and Flynn, G.C. (2011) High‐mannose glycans on the Fc region of therapeutic IgG antibodies increase serum clearance in humans. Glycobiology, 21, 949–959. 21421994 10.1093/glycob/cwr027

[pbi70176-bib-0030] Göritzer, K. and Strasser, R. (2021) Glycosylation of Plant‐Produced Immunoglobulins. Experientia Suppl. 112, 519–543. 10.1007/978-3-030-76912-3_16 34687021

[pbi70176-bib-0033] Göritzer, K. , Goet, I. , Duric, S. , Maresch, D. , Altmann, F. , Obinger, C. and Strasser, R. (2020) Efficient *N*‐glycosylation of the heavy chain tailpiece promotes the formation of plant‐produced dimeric IgA. Front. Chem. 8, 346. 32426328 10.3389/fchem.2020.00346 PMC7212365

[pbi70176-bib-0031] Göritzer, K. , Maresch, D. , Altmann, F. , Obinger, C. and Strasser, R. (2017) Exploring site‐specific N‐glycosylation of HEK293 and plant‐produced human IgA isotypes. J. Proteome Res. 16, 2560–2570. 28516782 10.1021/acs.jproteome.7b00121 PMC5504489

[pbi70176-bib-0035] Göritzer, K. , Melnik, S. , Schwestka, J. , Arcalis, E. , Drapal, M. , Fraser, P. , Ma, J.K. *et al*. (2025) Enhancing quality and yield of recombinant secretory IgA antibodies in *Nicotiana benthamiana* by endoplasmic reticulum engineering. Plant Biotechnol. J. 23, 14576. 10.1111/pbi.14576 PMC11933863 39822055

[pbi70176-bib-0034] Göritzer, K. , Ruocco, V. , Vavra, U. , Izadi, S. , Bolanos‐Martinez, O.C. , Phetphoung, T. , Pisuttinusart, N. *et al*. (2024) Improving the N‐glycosylation occupancy of plant‐produced IgG1 by engineering the amino acid environment at Asn297. Front. Plant Sci. 15, 1531710. 39911658 10.3389/fpls.2024.1531710 PMC11794253

[pbi70176-bib-0032] Göritzer, K. , Turupcu, A. , Maresch, D. , Novak, J. , Altmann, F. , Oostenbrink, C. , Obinger, C. *et al*. (2019) Distinct Fcα receptor *N*‐glycans modulate the binding affinity to immunoglobulin A (IgA) antibodies. J. Biol. Chem. 294, 13995–14008. 31362986 10.1074/jbc.RA119.009954 PMC6755811

[pbi70176-bib-0036] Grandits, M. , Grünwald‐Gruber, C. , Gastine, S. , Standing, J.F. , Reljic, R. , Teh, A.Y. and Ma, J.K. (2023) Improving the efficacy of plant‐made anti‐HIV monoclonal antibodies for clinical use. Front. Plant Sci. 14, 1126470. 36923134 10.3389/fpls.2023.1126470 PMC10009187

[pbi70176-bib-0037] Grosse‐Holz, F. , Madeira, L. , Zahid, M.A. , Songer, M. , Kourelis, J. , Fesenko, M. , Ninck, S. *et al*. (2018) Three unrelated protease inhibitors enhance accumulation of pharmaceutical recombinant proteins in *Nicotiana benthamiana* . Plant Biotechnol. J. 16, 1797–1810. 29509983 10.1111/pbi.12916 PMC6131417

[pbi70176-bib-0038] Gupta, M.D. , Flaskamp, Y. , Roentgen, R. , Juergens, H. , Armero‐Gimenez, J. , Albrecht, F. , Hemmerich, J. *et al*. (2023) Scaling eukaryotic cell‐free protein synthesis achieved with the versatile and high‐yielding tobacco BY‐2 cell lysate. Biotechnol. Bioeng. 120, 2890–2906. 37376851 10.1002/bit.28461

[pbi70176-bib-0039] Hamel, L.P. , Comeau, M.A. , Tardif, R. , Poirier‐Gravel, F. , Pare, M.E. , Lavoie, P.O. , Goulet, M.C. *et al*. (2024) Heterologous expression of influenza haemagglutinin leads to early and transient activation of the unfolded protein response in *Nicotiana benthamiana* . Plant Biotechnol. J. 22, 1146–1163. 38038125 10.1111/pbi.14252 PMC11022800

[pbi70176-bib-0040] Hanson, S.R. , Culyba, E.K. , Hsu, T.L. , Wong, C.H. , Kelly, J.W. and Powers, E.T. (2009) The core trisaccharide of an N‐linked glycoprotein intrinsically accelerates folding and enhances stability. Proc. Natl. Acad. Sci. U. S. A. 106, 3131–3136. 19204290 10.1073/pnas.0810318105 PMC2651298

[pbi70176-bib-0041] Harmoko, R. , Yoo, J.Y. , Ko, K.S. , Ramasamy, N.K. , Hwang, B.Y. , Lee, E.J. , Kim, H.S. *et al*. (2016) N‐glycan containing a core α1,3‐fucose residue is required for basipetal auxin transport and gravitropic response in rice (*Oryza sativa*). New Phytol. 212, 108–122. 27241276 10.1111/nph.14031

[pbi70176-bib-0042] He, M. , Zhou, X. and Wang, X. (2024) Glycosylation: mechanisms, biological functions and clinical implications. Signal Transduct. Target. Ther. 9, 194. 39098853 10.1038/s41392-024-01886-1 PMC11298558

[pbi70176-bib-0043] Hebert, D.N. , Foellmer, B. and Helenius, A. (1996) Calnexin and calreticulin promote folding, delay oligomerization and suppress degradation of influenza hemagglutinin in microsomes. EMBO J. 15, 2961–2968. 8670797 PMC450237

[pbi70176-bib-0044] Helenius, A. and Aebi, M. (2004) Roles of N‐linked glycans in the endoplasmic reticulum. Annu. Rev. Biochem. 73, 1019–1049. 15189166 10.1146/annurev.biochem.73.011303.073752

[pbi70176-bib-0045] Hiatt, A. , Pauly, M. , Whaley, K. , Qiu, X. , Kobinger, G. and Zeitlin, L. (2015) The emergence of antibody therapies for Ebola. Hum. Antibodies 23, 49–56. 27472862 10.3233/HAB-150284

[pbi70176-bib-0046] Hou, H.W. , Bishop, C.A. , Huckauf, J. , Broer, I. , Klaus, S. , Nausch, H. and Buyel, J.F. (2022) Seed‐ and leaf‐based expression of FGF21‐transferrin fusion proteins for oral delivery and treatment of non‐alcoholic steatohepatitis. Front. Plant Sci. 13, 998596. 36247628 10.3389/fpls.2022.998596 PMC9557105

[pbi70176-bib-0047] Huang, C.C. , Venturi, M. , Majeed, S. , Moore, M.J. , Phogat, S. , Zhang, M.Y. , Dimitrov, D.S. *et al*. (2004) Structural basis of tyrosine sulfation and VH‐gene usage in antibodies that recognize the HIV type 1 coreceptor‐binding site on gp120. Proc. Natl. Acad. Sci. U. S. A. 101, 2706–2711. 14981267 10.1073/pnas.0308527100 PMC365685

[pbi70176-bib-0050] Izadi, S. , Gumpelmair, S. , Coelho, P. , Duarte, H.O. , Gomes, J. , Leitner, J. , Kunnummel, V. *et al*. (2024) Plant‐derived Durvalumab variants show efficient PD‐1/PD‐L1 blockade and therapeutically favourable FcR binding. Plant Biotechnol. J. 22, 1224–1237. 38050338 10.1111/pbi.14260 PMC11022803

[pbi70176-bib-0049] Izadi, S. , Kunnummel, V. , Steinkellner, H. , Werner, S. and Castilho, A. (2023b) Assessment of transient expression strategies to sialylate recombinant proteins in *N. benthamiana* . J. Biotechnol. 365, 48–53. 36805356 10.1016/j.jbiotec.2023.02.004

[pbi70176-bib-0048] Izadi, S. , Vavra, U. , Melnik, S. , Grünwald‐Gruber, C. , Föderl‐Hobenreich, E. , Sack, M. , Zatloukal, K. *et al*. (2023a) In planta deglycosylation improves the SARS‐CoV‐2 neutralization activity of recombinant ACE2‐Fc. Front. Bioeng. Biotechnol. 11, 1180044. 37207124 10.3389/fbioe.2023.1180044 PMC10190127

[pbi70176-bib-0051] Jansing, J. , Sack, M. , Augustine, S.M. , Fischer, R. and Bortesi, L. (2019) CRISPR/Cas9‐mediated knockout of six glycosyltransferase genes in *Nicotiana benthamiana* for the production of recombinant proteins lacking β‐1,2‐xylose and core α‐1,3‐fucose. Plant Biotechnol. J. 17, 350–361. 29969180 10.1111/pbi.12981 PMC6335070

[pbi70176-bib-0052] Jeong, I.S. , Lee, S. , Bonkhofer, F. , Tolley, J. , Fukudome, A. , Nagashima, Y. , May, K. *et al*. (2018) Purification and characterization of *Arabidopsis thaliana* oligosaccharyltransferase complexes from the native host: a protein super‐expression system for structural studies. Plant J. 94, 131–145. 29385647 10.1111/tpj.13847

[pbi70176-bib-0053] Jin, C. , Bencúrová, M. , Borth, N. , Ferko, B. , Jensen‐Jarolim, E. , Altmann, F. and Hantusch, B. (2006) Immunoglobulin G specifically binding plant N‐glycans with high affinity could be generated in rabbits but not in mice. Glycobiology, 16, 349–357. 16373330 10.1093/glycob/cwj071

[pbi70176-bib-0054] Ju, M.S. and Jung, S.T. (2014) Aglycosylated full‐length IgG antibodies: steps toward next‐generation immunotherapeutics. Curr. Opin. Biotechnol. 30, 128–139. 25035939 10.1016/j.copbio.2014.06.013

[pbi70176-bib-0055] Jung, J.W. , Zahmanova, G. , Minkov, I. and Lomonossoff, G.P. (2022) Plant‐based expression and characterization of SARS‐CoV‐2 virus‐like particles presenting a native spike protein. Plant Biotechnol. J. 20, 1363–1372. 35325498 10.1111/pbi.13813 PMC9115404

[pbi70176-bib-0056] Kallolimath, S. , Castilho, A. , Strasser, R. , Grünwald‐Gruber, C. , Altmann, F. , Strubl, S. , Galuska, C.E. *et al*. (2016) Engineering of complex protein sialylation in plants. Proc. Natl. Acad. Sci. U. S. A. 113, 9498–9503. 27444013 10.1073/pnas.1604371113 PMC5003249

[pbi70176-bib-0057] Kallolimath, S. , Gruber, C. , Steinkellner, H. and Castilho, A. (2018) Promoter Choice Impacts the Efficiency of Plant Glyco‐Engineering. Biotechnol. J. 13, 5169. 10.1002/biot.201700380 28755501

[pbi70176-bib-0058] Kallolimath, S. , Hackl, T. , Gahn, R. , Grünwald‐Gruber, C. , Zich, W. , Kogelmann, B. , Lux, A. *et al*. (2020) Expression Profiling and Glycan Engineering of IgG Subclass 1–4 in. Front. Bioeng. Biotechnol. 8, 825. 32793574 10.3389/fbioe.2020.00825 PMC7393800

[pbi70176-bib-0059] Kallolimath, S. , Sun, L. , Palt, R. , Stiasny, K. , Mayrhofer, P. , Gruber, C. , Kogelmann, B. *et al*. (2021) Highly active engineered IgG3 antibodies against SARS‐CoV‐2. Proc. Natl. Acad. Sci. U. S. A. 118, e2107249118. 34599091 10.1073/pnas.2107249118 PMC8545452

[pbi70176-bib-0060] Karnoup, A.S. , Turkelson, V. and Anderson, W.H. (2005) O‐linked glycosylation in maize‐expressed human IgA1. Glycobiology, 15, 965–981. 15901675 10.1093/glycob/cwi077

[pbi70176-bib-0061] Keshvari, T. , Melnik, S. , Sun, L. , Niazi, A. , Aram, F. , Moghadam, A. , Kogelmann, B. *et al*. (2024) Efficient expression of functionally active aflibercept with designed N‐glycans. Antibodies, 13, 29. 38651409 10.3390/antib13020029 PMC11036266

[pbi70176-bib-0063] Kogelmann, B. , Melnik, S. , Bogner, M. , Kallolimath, S. , Stoger, E. , Sun, L. , Strasser, R. *et al*. (2024a) A genome‐edited *N. benthamiana* line for industrial‐scale production of recombinant glycoproteins with targeted N‐glycosylation. Biotechnol. J. 19, e2300323. 37804142 10.1002/biot.202300323 PMC11475529

[pbi70176-bib-0064] Kogelmann, B. , Melnik, S. , Keshvari, T. , Bogner, M. , Lavoie, P.O. , MA, D.A. , Hermle, A. *et al*. (2024b) An industrial‐grade *Nicotiana benthamiana* line for the production of glycoproteins carrying fucose‐free galactosylated N‐glycans. N. Biotechnol. 85, 23–30. 39613154 10.1016/j.nbt.2024.11.007

[pbi70176-bib-0062] Kogelmann, B. , Palt, R. , Maresch, D. , Strasser, R. , Altmann, F. , Kallolimath, S. , Sun, L. *et al*. (2023) In planta expression of active bacterial GDP‐6‐deoxy‐d‐lyxo‐4‐hexulose reductase for glycan modulation. Plant Biotechnol. J. 21, 1929–1931. 37553797 10.1111/pbi.14131 PMC10502745

[pbi70176-bib-0065] König‐Beihammer, J. , Vavra, U. , Shin, Y.J. , Veit, C. , Grünwald‐Gruber, C. , Gillitschka, Y. , Huber, J. *et al*. (2022) In planta production of the receptor‐binding domain from SARS‐CoV‐2 with human blood group A glycan structures. Front. Chem. 9, 816544. 35178379 10.3389/fchem.2021.816544 PMC8846405

[pbi70176-bib-0066] Kriechbaum, R. , Ziaee, E. , Grünwald‐Gruber, C. , Buscaill, P. , van der Hoorn, R.A.L. and Castilho, A. (2020) BGAL1 depletion boosts the level of β‐galactosylation of N‐ and O‐glycans in *N. benthamiana* . Plant Biotechnol. J. 18, 1537–1549. 31837192 10.1111/pbi.13316 PMC7292537

[pbi70176-bib-0067] Loos, A. , Gach, J.S. , Hackl, T. , Maresch, D. , Henkel, T. , Porodko, A. , Bui‐Minh, D. *et al*. (2015) Glycan modulation and sulfoengineering of anti‐HIV‐1 monoclonal antibody PG9 in plants. Proc. Natl. Acad. Sci. U. S. A. 112, 12675–12680. 26417081 10.1073/pnas.1509090112 PMC4611627

[pbi70176-bib-0068] Mamedov, T. , Cicek, K. , Miura, K. , Gulec, B. , Akinci, E. , Mammadova, G. and Hasanova, G. (2019) A plant‐produced in vivo deglycosylated full‐length Pfs48/45 as a transmission‐blocking vaccine candidate against malaria. Sci. Rep. 9, 1–12. 31285498 10.1038/s41598-019-46375-6 PMC6614448

[pbi70176-bib-0069] Mamedov, T. , Yuksel, D. , Ilgın, M. , Gurbuzaslan, I. , Gulec, B. , Yetiskin, H. , Uygut, M.A. *et al*. (2021) Plant‐produced glycosylated and in vivo deglycosylated receptor binding domain proteins of SARS‐CoV‐2 induce potent neutralizing responses in mice. Viruses, 13, 1595. 34452461 10.3390/v13081595 PMC8402646

[pbi70176-bib-0073] Margolin, E. , Allen, J.D. , Verbeek, M. , Chapman, R. , Meyers, A. , van Diepen, M. , Ximba, P. *et al*. (2022b) Augmenting glycosylation‐directed folding pathways enhances the fidelity of HIV Env immunogen production in plants. Biotechnol. Bioeng. 119, 2919–2937. 35781691 10.1002/bit.28169 PMC9544252

[pbi70176-bib-0070] Margolin, E. , Oh, Y.J. , Verbeek, M. , Naude, J. , Ponndorf, D. , Meshcheriakova, Y.A. , Peyret, H. *et al*. (2020a) Co‐expression of human calreticulin significantly improves the production of HIV gp140 and other viral glycoproteins in plants. Plant Biotechnol. J. 18, 2109–2117. 32096288 10.1111/pbi.13369 PMC7540014

[pbi70176-bib-0074] Margolin, E. , Schafer, G. , Allen, J.D. , Gers, S. , Woodward, J. , Sutherland, A.D. , Blumenthal, M. *et al*. (2023) A plant‐produced SARS‐CoV‐2 spike protein elicits heterologous immunity in hamsters. Front. Plant Sci. 14, 1146234. 36959936 10.3389/fpls.2023.1146234 PMC10028082

[pbi70176-bib-0072] Margolin, E. , Verbeek, M. , de Moor, W. , Chapman, R. , Meyers, A. , Schäfer, G. , Williamson, A.L. *et al*. (2022a) Investigating constraints along the plant secretory pathway to improve production of a SARS‐CoV‐2 spike vaccine candidate. Front. Plant Sci. 12, 798822. 35058959 10.3389/fpls.2021.798822 PMC8764404

[pbi70176-bib-0071] Margolin, E.A. , Strasser, R. , Chapman, R. , Williamson, A.L. , Rybicki, E.P. and Meyers, A.E. (2020b) Engineering the plant secretory pathway for the production of next‐generation pharmaceuticals. Trends Biotechnol. 38, 1034–1044. 32818443 10.1016/j.tibtech.2020.03.004

[pbi70176-bib-0075] Menacho‐Melgar, R. , Decker, J.S. , Hennigan, J.N. and Lynch, M.D. (2019) A review of lipidation in the development of advanced protein and peptide therapeutics. J. Control. Release, 295, 1–12. 30579981 10.1016/j.jconrel.2018.12.032 PMC7520907

[pbi70176-bib-0076] Meng, H. , Jain, S. , Lockshin, C. , Shaligram, U. , Martinez, J. , Genkin, D. , Hill, D.B. *et al*. (2018) Clinical Application of Polysialylated Deoxyribonuclease and Erythropoietin. Recent Pat. Drug Deliv. Formul. 12, 212–222. 30019653 10.2174/1872211312666180717164758

[pbi70176-bib-0077] Meuris, L. , Santens, F. , Elson, G. , Festjens, N. , Boone, M. , Dos Santos, A. , Devos, S. *et al*. (2014) GlycoDelete engineering of mammalian cells simplifies N‐glycosylation of recombinant proteins. Nat. Biotechnol. 32, 485–489. 24752077 10.1038/nbt.2885 PMC7039703

[pbi70176-bib-0078] Mimura, Y. , Mimura‐Kimura, Y. , Saldova, R. , Rudd, P.M. and Jefferis, R. (2022) Enhanced immunomodulatory effect of intravenous immunoglobulin by Fc Galactosylation and Nonfucosylation. Front. Immunol. 3, 818382. 10.3389/fimmu.2022.818382 PMC8831331 35154135

[pbi70176-bib-0079] Mócsai, R. , Göritzer, K. , Stenitzer, D. , Maresch, D. , Strasser, R. and Altmann, F. (2021) Prolyl hydroxylase paralogs in *Nicotiana benthamiana* show high similarity with regard to substrate specificity. Front. Plant Sci. 12, 636597. 33737944 10.3389/fpls.2021.636597 PMC7960765

[pbi70176-bib-0080] Montero‐Morales, L. , Maresch, D. , Castilho, A. , Turupcu, A. , Ilieva, K.M. , Crescioli, S. , Karagiannis, S.N. *et al*. (2017) Recombinant plant‐derived human IgE glycoproteomics. J. Proteomics 161, 81–87. 28400175 10.1016/j.jprot.2017.04.002

[pbi70176-bib-0081] Moore, C.M. , Grandits, M. , Grünwald‐Gruber, C. , Altmann, F. , Kotouckova, M. , Teh, A.Y. and Ma, J.K. (2021) Characterisation of a highly potent and near pan‐neutralising anti‐HIV monoclonal antibody expressed in tobacco plants. Retrovirology, 18, 17. 34183026 10.1186/s12977-021-00560-6 PMC8240387

[pbi70176-bib-0082] Nguyen, K.D. , Kajiura, H. , Kamiya, R. , Yoshida, T. , Misaki, R. and Fujiyama, K. (2023) Production and N‐glycan engineering of Varlilumab in *Nicotiana benthamiana* . Front. Plant Sci. 14, 1215580. 37615027 10.3389/fpls.2023.1215580 PMC10442953

[pbi70176-bib-0083] Pantazica, A.M. , van Eerde, A. , Dobrica, M.O. , Caras, I. , Ionescu, I. , Costache, A. , Tucureanu, C. *et al*. (2023) The “humanized” N‐glycosylation pathway in CRISPR/Cas9‐edited *Nicotiana benthamiana* significantly enhances the immunogenicity of a S/preS1 Hepatitis B Virus antigen and the virus‐neutralizing antibody response in vaccinated mice. Plant Biotechnol. J. 21, 1176–1190. 36779605 10.1111/pbi.14028 PMC10214758

[pbi70176-bib-0084] Parodi, A.J. (2000) Protein glucosylation and its role in protein folding. Annu. Rev. Biochem. 69, 69–93. 10966453 10.1146/annurev.biochem.69.1.69

[pbi70176-bib-0085] Pedersen, C.T. , Loke, I. , Lorentzen, A. , Wolf, S. , Kamble, M. , Kristensen, S.K. , Munch, D. *et al*. (2017) N‐glycan maturation mutants in *Lotus japonicus* for basic and applied glycoprotein research. Plant J. 91, 394–407. 28407380 10.1111/tpj.13570

[pbi70176-bib-0086] Petersen, B.L. , MacAlister, C.A. and Ulvskov, P. (2021) Plant protein O‐arabinosylation. Front. Plant Sci. 12, 5698. 10.3389/fpls.2021.645219 PMC8012813 33815452

[pbi70176-bib-0087] Pinkhasov, J. , Alvarez, M.L. , Rigano, M.M. , Piensook, K. , Larios, D. , Pabst, M. , Grass, J. *et al*. (2011) Recombinant plant‐expressed tumour‐associated MUC1 peptide is immunogenic and capable of breaking tolerance in MUC1.Tg mice. Plant Biotechnol. J. 9, 991–1001. 21740504 10.1111/j.1467-7652.2011.00614.x

[pbi70176-bib-0088] Piron, R. , Santens, F. , De Paepe, A. , Depicker, A. and Callewaert, N. (2015) Using GlycoDelete to produce proteins lacking plant‐specific N‐glycan modification in seeds. Nat. Biotechnol. 33, 1135–1137. 26544140 10.1038/nbt.3359

[pbi70176-bib-0089] Pisuttinusart, N. , Rattanapisit, K. , Srisaowakarn, C. , Thitithanyanont, A. , Strasser, R. , Shanmugaraj, B. and Phoolcharoen, W. (2024) Neutralizing activity of anti‐respiratory syncytial virus monoclonal antibody produced in *Nicotiana benthamiana* . Hum. Vaccin. Immunother. 20, 2327142. 38508690 10.1080/21645515.2024.2327142 PMC10956629

[pbi70176-bib-0090] Puchol Tarazona, A.A. , Maresch, D. , Grill, A. , Bakalarz, J. , Torres Acosta, J.A. , Castilho, A. , Steinkellner, H. *et al*. (2021) Identification of two subtilisin‐like serine proteases engaged in the degradation of recombinant proteins in *Nicotiana benthamiana* . FEBS Lett. 595, 379–388. 33263189 10.1002/1873-3468.14014 PMC8221030

[pbi70176-bib-0091] Qiu, X. , Wong, G. , Audet, J. , Bello, A. , Fernando, L. , Alimonti, J.B. , Fausther‐Bovendo, H. *et al*. (2014) Reversion of advanced Ebola virus disease in nonhuman primates with ZMapp. Nature, 514, 47–53. 25171469 10.1038/nature13777 PMC4214273

[pbi70176-bib-0092] Ramirez‐Alanis, I.A. , Renaud, J.B. , Garcia‐Lara, S. , Menassa, R. and Cardineau, G.A. (2018) Transient co‐expression with three O‐glycosylation enzymes allows production of GalNAc‐O‐glycosylated granulocyte‐colony stimulating factor in *N. benthamiana* . Plant Methods, 14, 98. 30410568 10.1186/s13007-018-0363-y PMC6219069

[pbi70176-bib-0093] Ramírez‐Andersen, H.S. , Behrens, C. , Buchardt, J. , Fels, J.J. , Folkesson, C.G. , Jianhe, C. , Nørskov‐Lauritsen, L. *et al*. (2018) Long‐acting human growth hormone analogue by noncovalent albumin binding. Bioconjug. Chem. 29, 3129–3143. 30168709 10.1021/acs.bioconjchem.8b00463

[pbi70176-bib-0095] Reusch, D. and Tejada, M.L. (2015) Fc glycans of therapeutic antibodies as critical quality attributes. Glycobiology 25, 1325–1334. 26263923 10.1093/glycob/cwv065 PMC4634315

[pbi70176-bib-0096] Ridgley, L.A. , Falci Finardi, N. , Gengenbach, B.B. , Opdensteinen, P. , Croxford, Z. , Ma, J.K. , Bodman‐Smith, M. *et al*. (2023) Killer to cure: expression and production costs calculation of tobacco plant‐made cancer‐immune checkpoint inhibitors. Plant Biotechnol. J. 21, 1254–1269. 36811226 10.1111/pbi.14034 PMC10214761

[pbi70176-bib-0097] Rosenberg, Y.J. , Jiang, X. , Lees, J.P. , Urban, L.A. , Mao, L. and Sack, M. (2022) Enhanced HIV SOSIP envelope yields in plants through transient co‐expression of peptidyl‐prolyl isomerase B and calreticulin chaperones and ER targeting. Sci. Rep. 12, 10027. 35705669 10.1038/s41598-022-14075-3 PMC9200074

[pbi70176-bib-0098] Ruiz‐Canada, C. , Kelleher, D.J. and Gilmore, R. (2009) Cotranslational and posttranslational N‐glycosylation of polypeptides by distinct mammalian OST isoforms. Cell, 136, 272–283. 19167329 10.1016/j.cell.2008.11.047 PMC2859625

[pbi70176-bib-0099] Ruocco, V. , Grünwald‐Gruber, C. , Rad, B. , Tscheliessnig, R. , Hammel, M. and Strasser, R. (2024) Effects of N‐glycans on the structure of human IgA2. Front. Mol. Biosci. 11, 1390659. 38645274 10.3389/fmolb.2024.1390659 PMC11026580

[pbi70176-bib-0100] Schachner, L.F. , Mullen, C. , Phung, W. , Hinkle, J.D. , Beardsley, M.I. , Bentley, T. , Day, P. *et al*. (2024) Exposing the molecular heterogeneity of glycosylated biotherapeutics. Nat. Commun. 15, 3259. 38627419 10.1038/s41467-024-47693-8 PMC11021452

[pbi70176-bib-0101] Schnaar, R.L. , Gerardy‐Schahn, R. and Hildebrandt, H. (2014) Sialic acids in the brain: gangliosides and polysialic acid in nervous system development, stability, disease, and regeneration. Physiol. Rev. 94, 461–518. 24692354 10.1152/physrev.00033.2013 PMC4044301

[pbi70176-bib-0102] Schneider, J. , Castilho, A. , Pabst, M. , Altmann, F. , Gruber, C. , Strasser, R. , Gattinger, P. *et al*. (2015) Characterization of plants expressing the human β1,4‐galactosyltrasferase gene. Plant Physiol. Biochem. 92, 39–47. 25900423 10.1016/j.plaphy.2015.04.010 PMC4451504

[pbi70176-bib-0103] Schwestka, J. , König‐Beihammer, J. , Shin, Y.J. , Vavra, U. , Kienzl, N.F. , Grünwald‐Gruber, C. , Maresch, D. *et al*. (2021) Impact of specific *N*‐glycan modifications on the use of plant‐produced SARS‐CoV‐2 antigens in serological assays. Front. Plant Sci. 12, 747500. 34646292 10.3389/fpls.2021.747500 PMC8503525

[pbi70176-bib-0104] Schwestka, J. , Zeh, L. , Tschofen, M. , Schubert, F. , Arcalis, E. , Esteve‐Gasent, M. , Pedrazzini, E. *et al*. (2023) Generation of multi‐layered protein bodies in *N. benthamiana* for the encapsulation of vaccine antigens. Front. Plant Sci. 14, 1109270. 36733717 10.3389/fpls.2023.1109270 PMC9887037

[pbi70176-bib-0105] Shields, R.L. , Lai, J. , Keck, R. , O'Connell, L.Y. , Hong, K. , Meng, Y.G. , Weikert, S.H. *et al*. (2002) Lack of fucose on human IgG1 N‐linked oligosaccharide improves binding to human Fcgamma RIII and antibody‐dependent cellular toxicity. J. Biol. Chem. 277, 26733–26740. 11986321 10.1074/jbc.M202069200

[pbi70176-bib-0106] Shin, Y.J. , Castilho, A. , Dicker, M. , Sádio, F. , Vavra, U. , Grünwald‐Gruber, C. , Kwon, T.H. *et al*. (2017) Reduced paucimannosidic N‐glycan formation by suppression of a specific β‐hexosaminidase from *Nicotiana benthamiana* . Plant Biotechnol. J. 15, 197–206. 27421111 10.1111/pbi.12602 PMC5259580

[pbi70176-bib-0107] Shin, Y.J. , König‐Beihammer, J. , Vavra, U. , Schwestka, J. , Kienzl, N.F. , Klausberger, M. , Laurent, E. *et al*. (2021) N‐Glycosylation of the SARS‐CoV‐2 receptor binding domain is important for functional expression in plants. Front. Plant Sci. 12, 689104. 34211491 10.3389/fpls.2021.689104 PMC8239413

[pbi70176-bib-0108] Shinkawa, T. , Nakamura, K. , Yamane, N. , Shoji‐Hosaka, E. , Kanda, Y. , Sakurada, M. , Uchida, K. *et al*. (2003) The absence of fucose but not the presence of galactose or bisecting N‐acetylglucosamine of human IgG1 complex‐type oligosaccharides shows the critical role of enhancing antibody‐dependent cellular cytotoxicity. J. Biol. Chem. 278, 3466–3473. 12427744 10.1074/jbc.M210665200

[pbi70176-bib-0110] Singh, A.A. , Pillay, P. , Naicker, P. , Alexandre, K. , Malatji, K. , Mach, L. , Steinkellner, H. *et al*. (2022) Transient proteolysis reduction of *Nicotiana benthamiana*‐produced CAP256 broadly neutralizing antibodies using CRISPR/Cas9. Front. Plant Sci. 13, 953654. 36061808 10.3389/fpls.2022.953654 PMC9433777

[pbi70176-bib-0109] Singh, A.A. , Pooe, O. , Kwezi, L. , Lotter‐Stark, T. , Stoychev, S.H. , Alexandra, K. , Gerber, I. *et al*. (2020) Plant‐based production of highly potent anti‐HIV antibodies with engineered posttranslational modifications. Sci. Rep. 10, 6201. 32277089 10.1038/s41598-020-63052-1 PMC7148297

[pbi70176-bib-0111] Song, J.H. , Jang, S. , Choi, J.W. , Hwang, S. , Kim, K.H. , Kim, H.Y. , Park, S.C. *et al*. (2024) Characterization of site‐specific N‐ and O‐glycopeptides from recombinant spike and ACE2 glycoproteins using LC‐MS/MS analysis. Int. J. Mol. Sci. 25, 13649. 39769415 10.3390/ijms252413649 PMC11678118

[pbi70176-bib-0112] Srisangsung, T. , Phetphoung, T. , Manopwisedjaroen, S. , Rattanapisit, K. , Bulaon, C.J.I. , Thitithanyanont, A. , Limprasutr, V. *et al*. (2024) The impact of N‐glycans on the immune response of plant‐produced SARS‐CoV‐2 RBD‐Fc proteins. Biotechnol. Rep. (Amst.) 43, e00847. 39040987 10.1016/j.btre.2024.e00847 PMC11261281

[pbi70176-bib-0113] Stadlmann, J. , Pabst, M. , Kolarich, D. , Kunert, R. and Altmann, F. (2008) Analysis of immunoglobulin glycosylation by LC‐ESI‐MS of glycopeptides and oligosaccharides. Proteomics 8, 2858–2871. 18655055 10.1002/pmic.200700968

[pbi70176-bib-0114] Stelter, S. , Paul, M.J. , Teh, A.Y. , Grandits, M. , Altmann, F. , Vanier, J. , Bardor, M. *et al*. (2020) Engineering the interactions between a plant‐produced HIV antibody and human Fc receptors. Plant Biotechnol. J. 18, 402–414. 31301102 10.1111/pbi.13207 PMC6953194

[pbi70176-bib-0115] Strasser, R. (2016) Plant protein glycosylation. Glycobiology, 26, 926–939. 26911286 10.1093/glycob/cww023 PMC5045529

[pbi70176-bib-0117] Strasser, R. , Castilho, A. , Stadlmann, J. , Kunert, R. , Quendler, H. , Gattinger, P. , Jez, J. *et al*. (2009) Improved virus neutralization by plant‐produced anti‐HIV antibodies with a homogeneous {beta}1,4‐galactosylated N‐glycan profile. J. Biol. Chem. 284, 20479–20485. 19478090 10.1074/jbc.M109.014126 PMC2742812

[pbi70176-bib-0116] Strasser, R. , Stadlmann, J. , Schähs, M. , Stiegler, G. , Quendler, H. , Mach, L. , Glössl, J. *et al*. (2008) Generation of glyco‐engineered *Nicotiana benthamiana* for the production of monoclonal antibodies with a homogeneous human‐like N‐glycan structure. Plant Biotechnol. J. 6, 392–402. 18346095 10.1111/j.1467-7652.2008.00330.x

[pbi70176-bib-0118] Sun, H. , Jugler, C. , Nguyen, K. , Steinkellner, H. and Chen, Q. (2023a) The potency and synergy of plant‐made monoclonal antibodies against the BA.5 variant of SARS‐CoV‐2. Plant Biotechnol. J. 21, 463–465. 36519524 10.1111/pbi.13980 PMC9877843

[pbi70176-bib-0120] Sun, H. , Yang, M. , Lai, H. , Neupane, B. , Teh, A.Y. , Jugler, C. , Ma, J.K. *et al*. (2023c) A dual‐approach strategy to optimize the safety and efficacy of anti‐Zika virus monoclonal antibody therapeutics. Viruses, 15, 2598. 10.3390/v15051156 PMC10221487 37243242

[pbi70176-bib-0119] Sun, L. , Kallolimath, S. , Palt, R. , Eminger, F. , Strasser, R. and Steinkellner, H. (2023b) Codon optimization regulates IgG3 and IgM expression and glycosylation in *N. benthamiana* . Front. Bioeng. Biotechnol. 11, 1320586. 38125307 10.3389/fbioe.2023.1320586 PMC10731585

[pbi70176-bib-0121] Swope, K. , Morton, J. , Pogue, G.P. , Burden, L. , Partain, N. , Hume, S. , Shepherd, J. *et al*. (2022) Reproducibility and flexibility of monoclonal antibody production with *Nicotiana benthamiana* . MAbs, 14, 2013594. 35000569 10.1080/19420862.2021.2013594 PMC8744878

[pbi70176-bib-0123] Thompson, N. and Wakarchuk, W. (2022) O‐glycosylation and its role in therapeutic proteins. Biosci. Rep. 42, 94. 10.1042/BSR20220094 PMC9620488 36214107

[pbi70176-bib-0124] Tretter, V. , Altmann, F. , Kubelka, V. , März, L. and Becker, W. (1993) Fucose alpha 1,3‐linked to the core region of glycoprotein N‐glycans creates an important epitope for IgE from honeybee venom allergic individuals. Int. Arch. Allergy Immunol. 102, 259–266. 7693094 10.1159/000236534

[pbi70176-bib-0126] Uetz, P. , Göritzer, K. , Vergara, E. , Melnik, S. , Grünwald‐Gruber, C. , Figl, R. , Deghmane, A.E. *et al*. (2024) Implications of O‐glycan modifications in the hinge region of a plant‐produced SARS‐CoV‐2‐IgA antibody on functionality. Front. Bioeng. Biotechnol. 12, 1329018. 38511130 10.3389/fbioe.2024.1329018 PMC10953500

[pbi70176-bib-0125] Uetz, P. , Melnik, S. , Grünwald‐Gruber, C. , Strasser, R. and Stöger, E. (2022) CRISPR/Cas9‐mediated knockout of a prolyl‐4‐hydroxylase subfamily in *Nicotiana benthamiana* using DsRed2 for plant selection. Biotechnol. J. 17, e2100698. 35427441 10.1002/biot.202100698

[pbi70176-bib-0145] van der Kaaij, A. , Bunte, M.J.M , Nijhof, L. , Mokhtari, S. , Overmars, H. , Schots, A. *et al*. (2025) Identification of β‐galactosidases along the secretory pathway of Nicotiana benthamiana that collectively hamper engineering of galactose‐extended glycans on recombinant glycoproteins. Plant Biotechnol. J. 10.1111/pbi.70126 PMC12854890 40333706

[pbi70176-bib-0094] van Ree, R. , Cabanes‐Macheteau, M. , Akkerdaas, J. , Milazzo, J.P. , Loutelier‐Bourhis, C. , Rayon, C. , Villalba, M. *et al*. (2000) Beta(1,2)‐xylose and alpha(1,3)‐fucose residues have a strong contribution in IgE binding to plant glycoallergens. J. Biol. Chem. 275, 11451–11458. 10753962 10.1074/jbc.275.15.11451

[pbi70176-bib-0127] Velasquez, S.M. , Ricardi, M.M. , Dorosz, J.G. , Fernandez, P.V. , Nadra, A.D. , Pol‐Fachin, L. , Egelund, J. *et al*. (2011) O‐glycosylated cell wall proteins are essential in root hair growth. Science, 332, 1401–1403. 21680836 10.1126/science.1206657

[pbi70176-bib-0128] Vézina, L.P. , Faye, L. , Lerouge, P. , D'Aoust, M.A. , Marquet‐Blouin, E. , Burel, C. , Lavoie, P.O. *et al*. (2009) Transient co‐expression for fast and high‐yield production of antibodies with human‐like N‐glycans in plants. Plant Biotechnol. J. 7, 442–455. 19422604 10.1111/j.1467-7652.2009.00414.x

[pbi70176-bib-0129] Wada, R. , Matsui, M. and Kawasaki, N. (2019) Influence of N‐glycosylation on effector functions and thermal stability of glycoengineered IgG1 monoclonal antibody with homogeneous glycoforms. MAbs, 11, 350–372. 30466347 10.1080/19420862.2018.1551044 PMC6380427

[pbi70176-bib-0130] Wagner, N. , Musiychuk, K. , Shoji, Y. , Tottey, S. , Streatfield, S.J. , Fischer, R. and Yusibov, V. (2024) Basic leucine zipper transcription activators ‐ tools to improve production and quality of human erythropoietin in Nicotiana benthamiana. Biotechnol. J. 19, e2300715. 38797727 10.1002/biot.202300715

[pbi70176-bib-0131] Walsh, G. and Jefferis, R. (2006) Post‐translational modifications in the context of therapeutic proteins. Nat. Biotechnol. 24, 1241–1252. 17033665 10.1038/nbt1252

[pbi70176-bib-0132] Wandall, H.H. , Nielsen, M.A.I. , King‐Smith, S. , de Haan, N. and Bagdonaite, I. (2021) Global functions of O‐glycosylation: promises and challenges in O‐glycobiology. FEBS J. 288, 7183–7212. 34346177 10.1111/febs.16148

[pbi70176-bib-0133] Wang, T.T. and Ravetch, J.V. (2019) Functional diversification of IgGs through Fc glycosylation. J. Clin. Invest. 129, 3492–3498. 31478910 10.1172/JCI130029 PMC6715372

[pbi70176-bib-0134] Wang, X.J. , Gao, Y.P. , Lu, N.N. , Li, W.S. , Xu, J.F. , Ying, X.Y. , Wu, G. *et al*. (2016) Endogenous Polysialic Acid Based Micelles for Calmodulin Antagonist Delivery against Vascular Dementia. ACS Appl. Mater. Interfaces, 8, 35045–35058. 27750011 10.1021/acsami.6b13052

[pbi70176-bib-0135] Watanabe, Y. , Bowden, T.A. , Wilson, I.A. and Crispin, M. (2019) Exploitation of glycosylation in enveloped virus pathobiology. Biochim. Biophys. Acta Gen. Sub. 1863, 1480–1497. 10.1016/j.bbagen.2019.05.012 PMC6686077 31121217

[pbi70176-bib-0137] Wilbers, R.H. , Westerhof, L.B. , van Noort, K. , Obieglo, K. , Driessen, N.N. , Everts, B. , Gringhuis, S.I. *et al*. (2017) Production and glyco‐engineering of immunomodulatory helminth glycoproteins in plants. Sci. Rep. 7, 45910. 28393916 10.1038/srep45910 PMC5385521

[pbi70176-bib-0136] Wilbers, R.H. , Westerhof, L.B. , van Raaij, D.R. , van Adrichem, M. , Prakasa, A.D. , Lozano‐Torres, J.L. , Bakker, J. *et al*. (2016) Co‐expression of the protease furin in *Nicotiana benthamiana* leads to efficient processing of latent transforming growth factor‐beta1 into a biologically active protein. Plant Biotechnol. J. 14, 1695–1704. 26834022 10.1111/pbi.12530 PMC5067602

[pbi70176-bib-0138] Wilson, I. , Zeleny, R. , Kolarich, D. , Staudacher, E. , Stroop, C. , Kamerling, J. and Altmann, F. (2001) Analysis of Asn‐linked glycans from vegetable foodstuffs: widespread occurrence of Lewis a, core alpha1,3‐linked fucose and xylose substitutions. Glycobiology, 11, 261–274. 11358875 10.1093/glycob/11.4.261

[pbi70176-bib-0140] Yang, M. , Sun, H. , Lai, H. , Neupane, B. , Bai, F. , Steinkellner, H. and Chen, Q. (2023) Plant‐produced anti‐Zika virus monoclonal antibody glycovariant exhibits abrogated antibody‐dependent enhancement of infection. Vaccines, 11, 755. 37112665 10.3390/vaccines11040755 PMC10144123

[pbi70176-bib-0139] Yang, Z. , Drew, D.P. , Jørgensen, B. , Mandel, U. , Bach, S.S. , Ulvskov, P. , Levery, S.B. *et al*. (2012) Engineering mammalian mucin‐type O‐glycosylation in plants. J. Biol. Chem. 287, 11911–11923. 22334671 10.1074/jbc.M111.312918 PMC3320939

[pbi70176-bib-0141] Yuan, Y. , Li, P. , Li, J. , Zhao, Q. , Chang, Y. and He, X. (2024) Protein lipidation in health and disease: molecular basis, physiological function and pathological implication. Signal Transduct. Target. Ther. 9, 60. 38485938 10.1038/s41392-024-01759-7 PMC10940682

[pbi70176-bib-0142] Zeitlin, L. , Pettitt, J. , Scully, C. , Bohorova, N. , Kim, D. , Pauly, M. , Hiatt, A. *et al*. (2011) Enhanced potency of a fucose‐free monoclonal antibody being developed as an Ebola virus immunoprotectant. Proc. Natl. Acad. Sci. U. S. A. 108, 20690–20694. 22143789 10.1073/pnas.1108360108 PMC3251097

[pbi70176-bib-0143] Zhang, Q. , Li, S. , He, L. and Feng, X. (2023) A brief review of polysialic acid‐based drug delivery systems. Int. J. Biol. Macromol. 230, 123151. 36610578 10.1016/j.ijbiomac.2023.123151

[pbi70176-bib-0144] Zwanenburg, L. , Borloo, J. , Decorte, B. , Bunte, M.J.M. , Mokhtari, S. , Serna, S. , Reichardt, N.C. *et al*. (2023) Plant‐based production of a protective vaccine antigen against the bovine parasitic nematode *Ostertagia ostertagi* . Sci. Rep. 13, 20488. 37993516 10.1038/s41598-023-47480-3 PMC10665551

